# Sex-Specific Association between Sodium Intake Estimated by 24-Hour Urinary Sodium Excretion and Nonalcoholic Fatty Liver Disease: The Community-Based Prospective Cohort Study

**DOI:** 10.3390/nu16040548

**Published:** 2024-02-16

**Authors:** Jihye Lee, Ju-Yeon Lee, Yun-Jung Yang

**Affiliations:** 1 Occupational Safety and Health Research Institute, Korea Occupational Safety and Health Agency, Ulsan 44429, Republic of Korea; neonsilver01@naver.com; 2 College of Medicine, Catholic Kwandong University, Gangneung-si 25601, Republic of Korea; jyeonlee@cku.ac.kr; 3 Department of Convergence Science, College of Medicine, Catholic Kwandong University International St. Mary’s Hospital, Incheon 22711, Republic of Korea

**Keywords:** sodium intake, nonalcoholic fatty liver disease, hepatic steatosis index, cohort study

## Abstract

Evidence for the association between high sodium intake and the onset of nonalcoholic fatty liver disease (NAFLD) is insufficient. This study examined the sex-specific association between sodium intake and the risk of NAFLD. This study included 2582 adults (aged 40–69 years; 1011 males and 1571 females). The total sodium excreted over 24 h was estimated from spot urine specimens using Tanaka’s equation. Based on these estimates, participants were categorized into three groups according to their 24-h urinary sodium excretion levels: lowest (T1), middle (T2), and highest (T3). In addition, the participants were divided into non-NAFLD (≤36) and NAFLD (>36) groups based on the hepatic steatosis index. During the follow-up period (14 years), NAFLD was observed in 551 participants. The estimated 24-h urinary sodium excretion levels were positively associated with the incidence of NAFLD in all subjects. Upon sex stratification, females in the T2 and T3 groups exhibited adjusted hazard ratios of 1.35 and 1.51, respectively, compared with the T1 group. However, a significant relationship was not observed in males. High intake of sodium, especially among females, may be an important factor contributing to the development of NAFLD. Individuals with high sodium intake should be appropriately counselled and monitored for the risk of NAFLD.

## 1. Introduction

Nonalcoholic fatty liver disease (NAFLD) is characterized by excessive lipid accumulation in hepatocytes that is not due to alcohol abuse or any other liver disease. This condition can progress from simple fat buildup to nonalcoholic steatohepatitis, which involves inflammation and liver cell damage, potentially leading to fibrosis, cirrhosis, and hepatocellular carcinoma if untreated [1,2]. An estimated 20–30% of adults worldwide are affected by NAFLD [3], which is commonly associated with cardiovascular diseases and conditions such as obesity, type 2 diabetes, insulin resistance, hypertension, dyslipidemia, and metabolic syndrome [4,5,6].

Various factors, including genetic susceptibility, metabolic abnormalities, environmental factors, and unhealthy lifestyle, have been suggested to affect NAFLD progression [3,5,7,8,9]. Several experimental and epidemiological studies have found that the consumption of high-fat or high-carbohydrate diets may influence the development of NAFLD [9,10,11]. Given the absence of any proven effective medical treatment for NAFLD, diet modification has been recommended as a potential approach to manage NAFLD [5].

Sodium chloride, commonly known as table salt, has long been employed as a natural food additive and is extensively used to augment the shelf life and enhance the palatability of foods [12]. The average daily sodium intake for adults worldwide is estimated to be around 4000 mg, equivalent to 10 g of salt per day [13]. Similarly, in Korea, the average sodium intake is 3600 mg/day in the general population [14], approximately twice the World Health Organization (WHO)’s recommended limit [15]. Excessive sodium intake may increase the risk of conditions such as obesity, dyslipidemia, insulin resistance, hypertension, type 2 diabetes, cardiovascular disease, stroke, and cancer [16,17,18,19,20,21], and the restriction of dietary sodium levels may prevent or suppress NAFLD progression in healthy adults. Recent studies have reported that high sodium intake may be associated with NAFLD development [22,23,24,25]. However, it remains challenging to assess the influence of NAFLD development on sodium intake levels because previous studies use cross-sectional datasets.

Therefore, this study investigated the sex-specific association between elevated sodium intake and the risk of NAFLD in Korean adults, utilizing data from a longitudinal prospective cohort study. Furthermore, we examined the association between the estimated 24-h urinary sodium levels and hepatic fibrosis in individuals with NAFLD.

## 2. Materials and Methods

### 2.1. Study Design and Participants

The Korean Genome and Epidemiology Study (KoGES) dataset was obtained from a large-scale study conducted by the Korea Center for Disease Control and Prevention. The dataset was established to assess the incidence and risk factors of non-communicable diseases and to investigate the genetic and environmental factors that may influence health outcomes in the Korean population [26]. The KoGES dataset, which has a longitudinal prospective cohort design, has recruited 10,030 adults aged 40–69 years from both urban (Ansan) and rural (Ansung) areas in South Korea. The study began with a baseline survey conducted between 2001 and 2002, during which data on participants’ health status, lifestyle habits, medical history, and other relevant information were collected [26]. After the baseline survey, the participants were followed up every two years.

**Figure 1:**
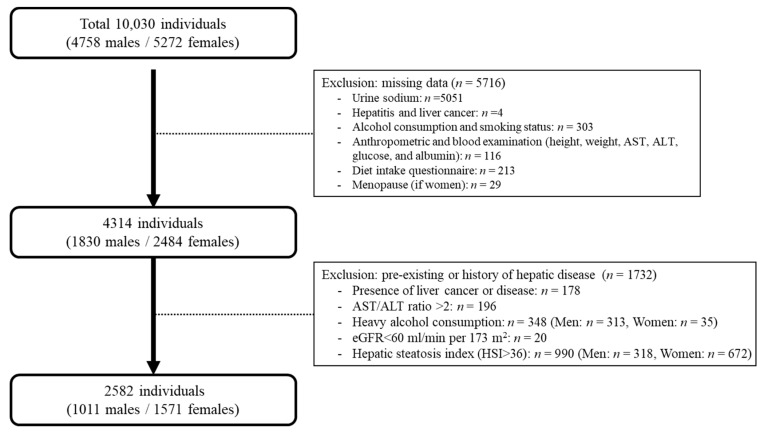
The selection procedure for the study population derived from the Ansung–Ansan cohort.

From the initial 10,030 adults, 5716 were excluded from the study: participants who did not undergo urinary sodium examination (*n* = 5051); those with missing information on hepatitis and liver cancer (*n* = 4); alcohol consumption and smoking status (*n* = 303); individuals lacking data on anthropometric measures and blood examination (including height, weight, alanine aminotransferase (ALT), aspartate aminotransferase (AST), glucose, blood pressure, and albumin; *n* = 116); those without baseline dietary habits information (*n* = 213); and females without menopausal status data (*n* = 29). Among the remaining participants, 1732 were excluded because of one of the following criteria: a self-reported history of liver cancer or liver disease (*n* = 178); excessive alcohol intake (>30 g/day in males [*n* = 313] and >20 g/day in females [*n* = 35]; *n* = 348); AST/ALT > 2, which can be an indicator of alcoholic liver disease (*n* = 196); pre-existing NAFLD based on hepatic steatosis index (HSI) > 36 (*n* = 990) and estimated glomerular filtration rate (eGFR) of <60 mL/min per 1.73 m^2^ (*n* = 20), which can be indicators of chronic kidney disease. A total of 2582 participants (*n* = 1011 males and *n* = 1571 females) were included in the analysis (Figure 1). The Institutional Review Board of the Catholic Kwandong University International St. Mary’s Hospital approved the study protocol (IS23EISI0081, 19 September 2023).

### 2.2. Estimation of 24-Hour Sodium Excretion

Sodium intake was estimated based on the value of 24-h urinary sodium levels. The estimation of 24-h urinary sodium level was calculated using Tanaka’s equation [27], which considers the values of sodium (Na) and creatinine (Cr) in spot urine samples. The estimated 24-h urinary sodium excretion (mmol/day) was calculated using the following formulas: (1) estimated Na (mmol/day) = 23 × (21.98 × XNa^0.392^); (2) XNa = {spot urine Na (mmol/L) / [10 × spot urine Cr (mg/dL)]} × predicted Cr (mg/day); (3) predicted Cr (mg/day) = −2.04 × age (yr) + 14.89 × weight (kg) + 6.14 × height (cm) − 2244.45. The estimated 24-h urinary sodium levels were categorized into the lowest (T1), middle (T2), and highest (T3) tertiles. T1 was chosen as a reference for analysis due to the absence of a cut-off value in 24-h urinary sodium levels.

### 2.3. Determination of NAFLD Based on Hepatic Steatosis Index (HSI)

To determine NAFLD status, the HSI, a simple and efficient screening tool, was used. Serum ALT and AST levels, body mass index (BMI), and diabetes mellitus (DM) status were utilized to calculate the HSI using the following formula [28]: HSI = 8 × ALT/AST + BMI (+2, if female; +2, if DM). The subjects were categorized into non-NAFLD (HSI ≤ 36) and NAFLD (HSI > 36) groups.

### 2.4. Determination of Hepatic Fibrosis Based on Fibrosis-4 (FIB-4)

To categorize subjects with hepatic fibrosis, the fibrosis-4 (FIB-4) score which is a simple noninvasive index was used [29]. In subjects with NAFLD based on HSI (HSI > 36), we evaluated the severity of liver disease. The formula used for calculation was as follows: age (years) × AST [U/L]/(platelets [10^9^/L] × (ALT [U/L])^1/2^). Hepatic fibrosis was determined in patients with FIB-4 ≥ 1.45. Additionally, the subjects with FIB-4 score < 1.45 were categorized as non-hepatic fibrosis.

### 2.5. Covariates

Demographic information, including age, drinking and smoking status, physical activity, medical history, and medication use, was obtained by a trained interviewer through a face-to-face survey. Height and weight measurements were taken with a digital stadiometer and a scale, respectively. After a 5 min rest period, blood pressure was measured in a seated position using a conventional mercury sphygmomanometer. Blood and urine samples were collected after fasting for at least 8 h. To evaluate the dietary intake of the participants, a semi-quantitative food frequency questionnaire (FFQ) comprising 103 items was specifically designed for KoGES. Detailed information on the questionnaires, physical examination, and FFQ has been described elsewhere [26].

Of the data obtained from the cohort study, we used age, drinking status, smoking status, and physical activity data from a questionnaire-based survey. Age was divided into two categories based on the median age. The consumption status of smoking and alcohol were categorized as never, former, or current. Physical activity intensity was divided into tertiles of the metabolic equivalent of task (MET) values (hour/week). The homeostatic model assessment of insulin resistance (HOMA-IR) was used to calculate insulin resistance as {fasting insulin (mU/L) × fasting glucose (mmol/L)}/22.5. Kidney function was assessed by calculating the eGFR using the Modification of Diet in Renal Disease Study equation, the most commonly utilized formula for this purpose [30]. Hypertension was identified based on a self-reported history of hypertension, use of antihypertensive medication, and a systolic blood pressure of 140 mmHg or higher, or a diastolic blood pressure of 90 mmHg or higher. Hyperlipidemia was identified based on a self-reported history of hyperlipidemia, the use of anti-hyperlipidemia medication, high-density lipoprotein (HDL)-cholesterol levels of 40 mg/dL or lower, low density lipoprotein-cholesterol levels of 160 mg/dL or higher, or triglyceride levels of 240 mg/dL or higher. Diabetes mellitus (DM) was determined through self-reported history, use of DM medication, or fasting glucose level of 126 mg/dL or above. Daily energy (kcal), protein (g), fat (g), and carbohydrate (g) intakes, as well as biochemical parameters, including fasting plasma glucose, fasting insulin, triglycerides (TG), total cholesterol (T-Chol), ALT, AST, HDL, platelets, and albumin levels, were obtained from survey data.

### 2.6. Statistical Analysis

The liver is a sexually dimorphic organ that exhibits distinct responses to sex hormones. Strong sexual dimorphism has been observed in patients with NAFLD [31,32,33]. Thus, statistical analyses were performed and stratified according to sex.

To evaluate the normality of continuous variables, skewness and kurtosis tests were performed. For variables with a normal distribution, the data are presented as the mean and standard deviation (SD). For the variables that do not follow a normal distribution, the data are presented as the median and interquartile range (IQR). Categorical variables are presented as the number (*n*) and percentage (%). Baseline characteristics of data adhering to a normal distribution were examined using analysis of variance (ANOVA) for continuous variables and Chi-squared tests for categorical variables. If variables were not normally distributed, the Kruskal–Wallis test was performed for continuous variables and Fisher’s exact test for categorical variables.

The differences between baseline and the onset of outcome or final follow-up were considered survival times. Survival time refers to the duration between baseline assessment and the occurrence of a specific outcome or completion of the final follow-up. The incidence time was defined as the difference in duration between the dates of the initial and final participation. The data for cases in which patients were lost to follow-up or died during the study period were considered censored. The Cox proportional hazards regression model was used to estimate the longitudinal relationship between 24-h sodium excretion and NAFLD risk, as well as to evaluate the potential effects of estimated 24-h sodium excretion levels on the incidence of hepatic fibrosis, before and after adjusting for covariates. In the multivariate analysis, variables that exhibited *p*-values less than 0.10 in the univariate analysis were incorporated. For the multivariate analysis, AST, ALT, BMI, and DM were excluded due to their use in NAFLD classification, and for hepatic fibrosis risk evaluation, platelets and age were also omitted. The Kaplan–Meier method, along with the log-rank test, were employed to compare the cumulative rates of NAFLD incidents (including hepatic fibrosis) based on the tertiles of 24-h sodium excretion.

All statistical analyses were conducted using STATA (version 18.0 StataCorp LP, College Station, TX, USA). Statistical significance was set at *p* < 0.05.

## 3. Results

### 3.1. The Distribution of Estimated 24-Hour Urinary Sodium Levels

The average concentration of the estimated 24-h urinary sodium was 161.69 ± 35.57 mmol/day in all subjects (Table 1). After sex stratification, the mean levels of estimated 24-h sodium were 162.03 ± 35.47 mmol/day in males and 161.48 ± 35.65 mmol/day in females.

### 3.2. Baseline Characteristics of Study Population

The enrolled participants (*n* = 2582) were classified into tertiles according to the estimated concentrations of 24-h urinary sodium excretion (Table 2). There was significant difference among the groups regarding age, BMI, smoking status, TG levels, urine potassium levels, hypertension status, postmenopausal status, and total energy intake, as well as the intake of protein, fat, and carbohydrates, based on the estimated 24-h urinary sodium intake levels of the overall participants.

The baseline characteristics of the 1011 males who were classified into tertiles according to the estimated 24-h urinary sodium excretion (Table S1). The median and IQR of 24-h urinary sodium excretion were 129.63 (117.16–138.36) mmol/day in the T1, 160.99 (153.54–168.07) mmol/day in the T2, and 192.54 (182.89–206.84) mmol/day in the T3 group (*p* < 0.001). Age, BMI, TG levels, urine potassium levels, and eGFR were significantly different among the groups (*p* = 0.002, *p* < 0.001, *p* < 0.014, *p* < 0.001, and *p* < 0.001, respectively). The proportion of current smokers and subjects with hypertension was significantly different among the groups (both *p* < 0.001). Total energy, protein, fat, and carbohydrate intakes were significantly different between the groups (*p* < 0.001, *p* < 0.001, *p* = 0.001, and *p* < 0.001, respectively).

The baseline characteristics of the 1571 female participants across the 24-h urinary sodium excretion tertiles are listed in Table S2. The median and IQR of 24-h urinary sodium excretion was 129.03 (114.40–138.75) mmol/day in the T1, 160.20 (152.68–166.82) mmol/day in the T2, and 193.14 (182.58–210.16) mmol/day in the T3 group (*p* < 0.001). Age, BMI, physical activity, ALT level, TG level, T-Chol level, urine potassium levels, and HOMA-IR were significantly different between groups (*p* < 0.001, *p* < 0.001, *p* = 0.029, *p* = 0.028, *p* < 0.001, *p* = 0.004, *p* < 0.001, and *p* = 0.016, respectively). Additionally, in the T3 group, the percentage of female participants with hypertension and those who were postmenopausal was higher than in the T1 group (*p* = 0.002 and *p* = 0.001, respectively). Total protein and carbohydrate intake were significantly different between the groups (*p* = 0.005 and *p* < 0.001, respectively).

### 3.3. Longitudinal Relationship between Estimated 24-Hour Urinary Sodium Excretion and Incidence of NAFLD

The risk of NAFLD incidence was analyzed based on the 24-h urinary sodium excretion of participants (Table 3). The number of NAFLD incidents significantly differed among the groups (*p* < 0.001). The HRs (95% CI) for NAFLD incidence increased with the elevation of estimated 24-h urinary sodium excretion levels (*p* for trend < 0.001). After adjusting the covariates, the HRs (95% CI) for NAFLD incidence in the T2 and T3 groups were 1.31 (1.05–1.64) and 1.54 (1.24–1.92), respectively (*p* for trend < 0.001). Age, smoking status, serum TG levels, HOMA-IR, eGFR, and hypertension were significantly associated with the incidence of NAFLD (Table S3).

After sex-stratifying the risk of NAFLD incidence based on estimated 24-h urinary sodium excretion, approximately 14.54% (49/337) of the T1, 17.21% (58/337) of the T2, and 19.58% (66/337) of the T3 group developed NAFLD in males (*p* = 0.174). The risk of developing NAFLD tended to be higher in the T2 and T3 groups (HR 1.18 [95% CI 0.80–1.72] and HR 1.41 [95% CI 0.98–2.04], respectively); however, statistical significance was not observed (*p* for trend = 0.064). After adjusting for potential confounding factors, the HRs (95% CI) for NAFLD incidence were significantly increased in T3 (1.50 [1.02–2.19]) compared with that in the T1 group (*p* for trend < 0.001). Age, TG, HOMA-IR, and eGFR in males were detected as potential confounders in the multivariate analysis (Table S4).

In addition, the incidence of NAFLD in females significantly increased following the increase in the estimated 24-h urinary sodium excretion from 17.17% (90/524) in the T1 and 23.66% (124/524) in the T2 groups to 26.19% (137/523) in the T3 group (*p* = 0.001). Based on the crude analysis, the risks of NAFLD development were significantly higher in the T2 with an HR of 1.40 (95% CI 1.07–1.84) and the T3 group with an HR of 1.62 (95% CI 1.24–2.12) than in the T1 group (*p* for trend < 0.001). Even when covariates were adjusted, the HRs (95% CI) for NAFLD development in the T2 and T3 groups were 1.35 (1.03–1.78) and 1.51 (1.15–1.98), respectively, which were higher than the HR (95% CI) in the T1 group, and the trend was significantly linear (*p* for trend = 0.003). Serum TG levels were identified as a potential risk factor for NAFLD incidence in females (Table S5).

**Figure 2:**
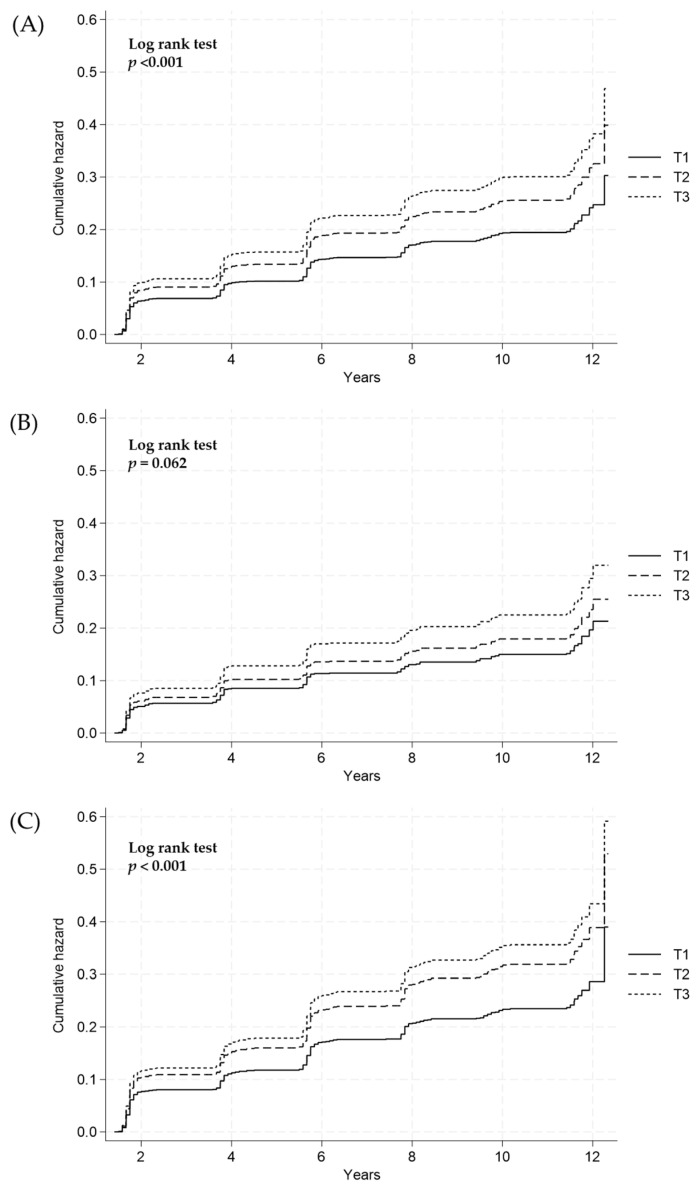
Cumulative incidence of NAFLD according to the 24-h sodium excretion tertiles (T) in (**A**) all subjects, (**B**) males, and (**C**) females using the Cox proportional hazard model. NAFLD, nonalcoholic fatty liver disease; T1, lowest tertile; T2, middle tertile; T3, highest tertile.

The cumulative incidence of NAFLD, based on the estimated 24-h urinary sodium excretion, was compared across groups using the log-rank test. The overall risk of NAFLD incidence significantly increased with the increase in 24-h urinary sodium excretion (log–rank test, *p* < 0.001; Figure 2A). After sex stratification, the estimated 24-h urinary sodium excretion was not significantly associated with the risk of NAFLD incidence in males (log-rank test, *p* = 0.062; Figure 2B). However, in females, a significant association was observed between the estimated 24-h urinary sodium excretion and the risk of NAFLD (log-rank test, *p* < 0.001; Figure 2C).

### 3.4. Longitudinal Relationship between Estimated 24-Hour Urinary Sodium Excretion and Incidence of Hepatic Fibrosis in Subjects with NAFLD

We classified the participants with NAFLD (HSI > 36) to identify hepatic fibrosis based on the FIB-4 score (FIB-4 ≥ 1.45). The HRs (95% CI) of hepatic fibrosis in all subjects were 2.25 (1.35–3.77) in T2 and 2.37 (1.42–3.95) in T3 compared with T1 (Table 4). The significant relationship between estimated 24-h urinary sodium excretion levels and the risk of hepatic fibrosis was also demonstrated after adjusting for confounding factors. Furthermore, in the multivariate analysis, an increase in eGFR was identified as a protective factor, whereas serum TG levels and hypertension were recognized as possible risk factors in the development of hepatic fibrosis associated with sodium intake (Table S6).

After stratifying by sex, the incidence of hepatic fibrosis in males might not be associated with the estimated 24-h urinary sodium excretion levels in both crude and multivariate analysis (*p* for trend = 0.083 and 0.073, respectively). Elevated eGFR might act as a protective factor; however, serum TG levels was detected as a potential risk factor in the incidence of hepatic fibrosis, which resulted from an increase in the estimated 24-h urinary sodium excretion levels in males (Table S7). Meanwhile, in females, a significant increase in HRs (95% CI) of hepatic fibrosis both T2 and T3 was observed compared with T1 (2.54 [1.30–4.95] and 2.61 [1.34–5.09], respectively). The significant relationship between the estimated 24-h urinary sodium excretion levels and the incidence risk of hepatic fibrosis was maintained even after adjusting for potential risk factors. Hypertension might be a potential risk factor for the development of hepatic fibrosis in females, whereas a higher eGFR might reduce the risk of developing this condition (Table S8).

**Figure 3:**
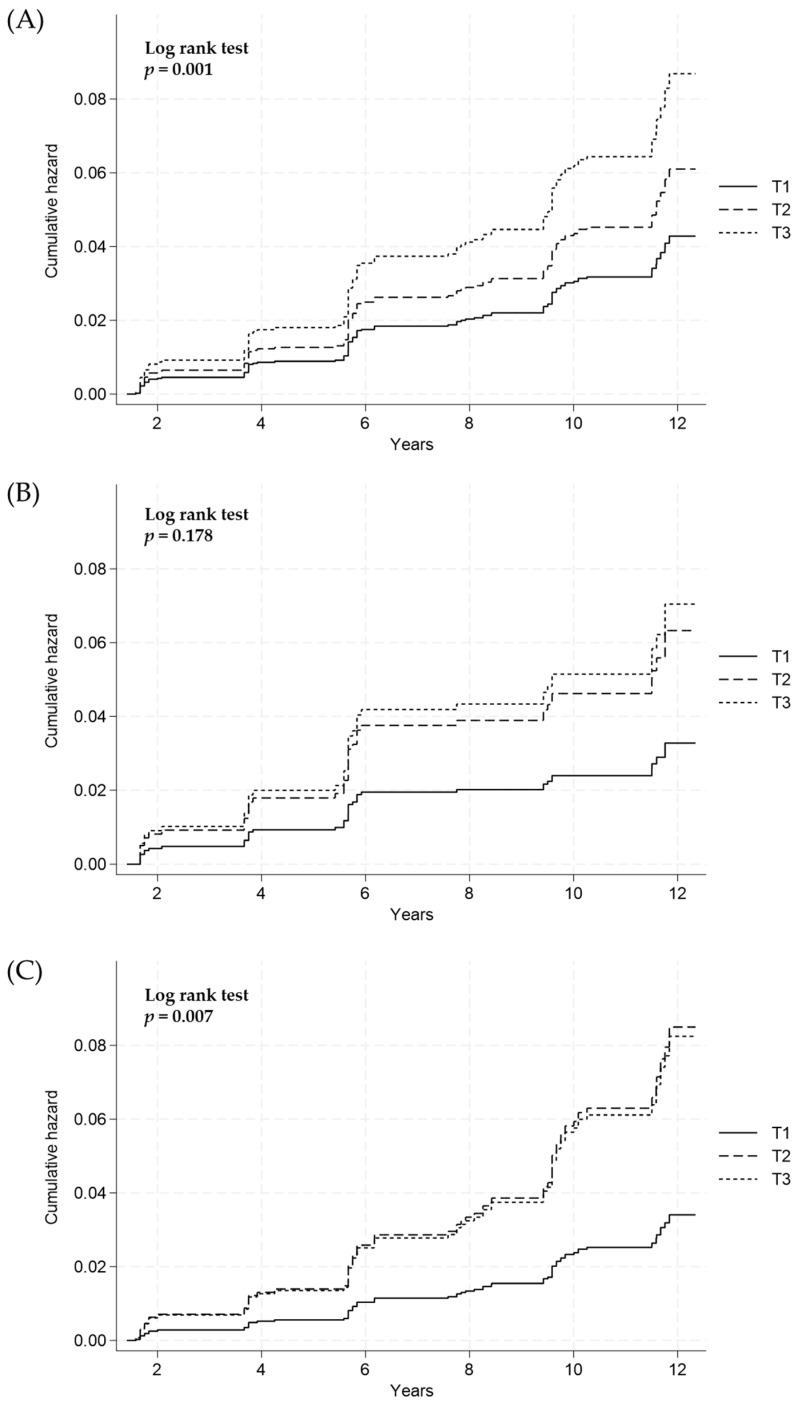
Cumulative incidence of hepatic fibrosis according to 24-h sodium excretion tertiles (T) in (**A**) all subjects, (**B**) males, and (**C**) females using the Cox proportional hazard model. T1, lowest tertile; T2, middle tertile; T3, highest tertile.

The cumulative incidence of hepatic fibrosis according to the estimated 24-h urinary sodium excretion was compared between the groups using the log-rank test. In overall subjects, the risks of the development of hepatic fibrosis among participants with NAFLD were significantly increased with the elevation of the estimated 24-h urinary sodium excretion levels (log-rank test, *p* = 0.001; Figure 3A). After sex stratification, no significant association between the estimated 24-h urinary sodium excretion and the risk of hepatic fibrosis in males was observed (log-rank test, *p* = 0.178; Figure 3B), whereas a statistical significance was found in females (log-rank test, *p* =0.007; Figure 3C).

## 4. Dishcussion

This study assessed the relationship between sodium intake levels, estimated by 24-h urinary sodium excretion, and NAFLD, as determined by the HSI score, in the Korean population. A significant association between high sodium intake and the risk of NAFLD was observed even after adjusting for potential confounding factors. This relationship remained strong in females but not in males when analyzed separately. Additionally, higher sodium intake was associated with an increased risk of hepatic fibrosis in all subjects. This significant association was only detected in females after stratification by sex. Based on our results, excessively high levels of sodium intake, particularly in females, could elevate the risk of developing NAFLD.

Salt, predominantly in the form of sodium chloride, is a ubiquitous component of modern diets and is largely responsible for the widespread consumption of processed and fast foods [13]. In Korea, an extensive range of fermented foods, such as kimchi, soy sauce, and soybean paste, constitute primary reservoirs of dietary sodium [34]. Sodium intake may increase the risk of obesity and metabolic disorders including hypertension, dyslipidemia, insulin resistance, hypertension, and type 2 diabetes [16,17,18,19,20,21]. As these metabolic disorders are related to NAFLD, there has been a growing interest in the influence of sodium intake on the risk of NAFLD development and progression. Previously, it has been reported that consuming high levels of sodium is independently associated with an increased risk of NAFLD and liver fibrosis among the general adult population [22,23,24]. Furthermore, a causal relationship between high sodium intake and the risk of NAFLD, after adjusting for potential confounders, was observed [35,36]. However, a study found no association between sodium intake and NAFLD risk in elderly patients [37]. This discrepancy might be due to differences in the sodium intake classification methods; in the latter study, classification was based on the presence of sodium consumption rather than on the quantity consumed.

In addition, the pathophysiology of the liver is sexually dimorphic, establishing NAFLD as a condition characterized by sexual dimorphism [31]. In general, the prevalence of NAFLD is higher in males than that in females [38,39], possibly due to differences in adipose distribution and metabolism, and hormonal factors such as estradiol, which might have protective effects on the liver [31,32,33]. Additionally, a gradually increasing prevalence of NAFLD has been observed in females but not in males [38]. Similar results have been reported in other cross-sectional and retrospective studies [23,39]. Our study also demonstrated a strong causal relationship between elevated sodium intake and NAFLD in females. In addition, females have a higher prevalence of NAFLD than males. Although at first sight, this finding may contradict the other finding that males are more susceptible to NAFLD than females, this gap is not surprising because of the reduction in estradiol levels in aged females. The levels of liver function enzymes, including AST, ALT, gamma-glutamylytransferase, and alkaline, were significantly decreased in females treated with hormone replacement therapy [40]. Females who experience hormonal changes with age may be more susceptible to NAFLD. In addition, elevated sodium intake may accelerate the progression of NAFLD in females.

The underlying mechanisms through which sodium intake influences NAFLD pathogenesis have not been determined. However, there are several possible mechanisms. One of the primary pathways currently explored is the role of sodium in blood pressure regulation [41,42]. Thus, prolonged elevation in blood pressure establishes a progressive correlation between increased dietary sodium content, arterial hypertension, and elevated stiffness of the large arteries.

A high dietary sodium intake triggers changes in the extracellular matrix of the arterial wall, contributing to arterial stiffening [41]. Considering that hypertension is a risk factor for NAFLD [3], this correlation implies a potential pathway. Another possible pathway is that high sodium intake leads to elevated leptin production, which can increase visceral adipose tissue and modify glucose and lipid metabolism, consequently leading to the accumulation of hepatic lipids (i.e., hepatic steatosis) [5]. Furthermore, sodium may affect insulin resistance, oxidative stress, and inflammation associated with NAFLD pathophysiology [5,42,43]. Elevated visceral adipose tissue causes insulin resistance and increases free fatty acids. This may result in hepatic steatosis and an environment rich in proinflammatory mediators, leading to cellular injury in the liver and beyond. As the underlying mechanisms are not yet clearly understood, further investigation is needed to shed light on this complex relationship.

Our study had several limitations. First, we employed surrogate markers, such as HSI for NAFLD diagnosis and FIB-4 for hepatic fibrosis, rather than hepatic imaging or biopsy. Abdominal ultrasonography and liver biopsy are more appropriate than the HSI and FIB-4 index. However, these methods do not apply to population-based large-scale studies due to cost and invasiveness issues. The HSI encompasses AST and ALT levels and BMI [28]. The sensitivity and specificity of HSI > 36 for NAFLD diagnosis are 93.1% (95% CI 92.1–94.1) and 93.1% (95% CI 92.0–94.0), respectively. In addition, FIB-4 is the most commonly used non-invasive index to assess hepatic fibrosis [29]. An FIB-4 value of <1.45 indicates a 90% negative predictive value, while >3.25 has a 65% positive predictive value for the diagnosis of hepatic fibrosis. We used a FIB-4 value of <1.45 as the cutoff to distinguish between participants with NAFLD but no hepatic fibrosis and those with NAFLD and hepatic fibrosis. Second, due to insufficient data on conditions such as autoimmune hepatitis, medication-induced hepatitis, or Wilson’s disease, the possibility of secondary fatty liver could not be entirely excluded. Nevertheless, we minimized this concern by excluding individuals with a history of liver disease, including hepatitis, alcoholic liver disease (i.e., AST/ALT > 2), and heavy alcohol consumption [44,45]. These exclusions are particularly relevant as they address a significant proportion of secondary fatty liver cases in Korea. Third, we estimated the 24-h urinary sodium excretion using spot urine samples from participants. The measurement of sodium excretion through a 24-h urine collection has emerged as a favored approach for evaluating dietary sodium intake in population surveys because of the challenges and inaccuracies associated with dietary recall. Nonetheless, the procedures for collecting 24-h urine samples are both expensive and inconvenient for study participants. As a result, the estimation of sodium excretion using spot urine specimens has gained widespread adoption for assessing individual sodium intake [27]. Fourth, the participants were only Korean; hence, it would be difficult to generalize our results to other ethnic populations. Fifth, due to the lack of information in KoGES, we could not evaluate factors such as diuretic usage, adrenal dysfunction, or urinary tract infections, all of which can influence Urinary sodium excretion [22].

Despite these limitations, this study possessed numerous strengths. First, this was the first study to suggest a causal relationship between high sodium intake and the risk of NAFLD, utilizing a large population-based prospective cohort study. Second, we considered variables for various nutrient intakes that could serve as potential confounding factors. Third, considering the sexual dimorphism in NAFLD, we analyzed sex stratification. Lastly, although the concentration of sodium intake in this study population was lower than that in the general Korean population, an association between sodium intake and the incidence of NAFLD was observed. This finding necessitates a further reduction in sodium intake, highlighting the fact that Koreans consume excessive amounts of sodium.

## 5. Conclusions

This study showed that elevated sodium intake may influence the increased risk of NAFLD in middle-aged and older Korean females. Despite subjects with NAFLD being classified using a surrogate index, HSI, our findings suggest that high sodium intake is a potential risk factor for NAFLD development. In addition, a significant association was found between sodium intake levels and the risk of developing hepatic fibrosis. Large-scale prospective studies are needed to clarify the potential impact of high sodium intake on NAFLD, especially in females. Furthermore, laboratory studies are crucial to identify the biological mechanisms underlying this association.

## Figures and Tables

**Table 1:** Distribution of 24-h urinary sodium excretion levels (mmol/day) in study participants.

	Mean	SD	Min	Percentile			Max
				25th	50th	75th	
All subjects (*n* = 2582)	161.69	35.57	62.72	138.63	160.42	182.62	391.32
Males (*n* = 1011)	162.03	35.47	64.04	138.36	160.99	182.89	373.08
Females (*n* = 1571)	161.48	35.65	62.72	138.74	160.20	182.58	391.32

SD, standard deviation; Min, minimum; Max, maximum.

**Table 2:** Baseline characteristics of study participants (*n* = 2582) according to 24-h urinary sodium excretion.

Characteristics	Tertiles of 24-Hour Urinary Sodium Excretion (mmol/Day)			*p*-Value
	T1 (*n* = 861)	T2 (*n* = 861)	T3 (*n* = 860)	
24-h urinary sodium, mmol/day	129.11 (115.53–138.63)	160.42 (152.95–167.25)	192.76 (182.63–208.43)	<0.001 ^†^
Age, yr	49 (43–59)	51 (44–61)	53 (45–61)	<0.001 ^†^
Gender (females), *n* (%)	529 (61.44)	531 (61.67)	511 (59.42)	0.574
BMI, kg/m^2^	22.91 (21.41–24.61)	23.53 (21.90–25.01)	23.89 (22.39–25.34)	<0.001 ^†^
Drinking status, *n* (%)				0.783
Never	489 (56.79)	472 (54.82)	487 (56.63)	
Former	58 (6.74)	66 (7.67)	54 (6.28)	
Current	314 (36.47)	323 (37.51)	319 (37.09)	
Smoking status, *n* (%)				0.002
Never	555 (64.46)	585 (67.94)	602 (70.00)	
Former	82 (9.52)	93 (10.80)	102 (11.86)	
Current	224 (26.02)	183 (21.25)	156 (18.14)	
Physical activity, MET-h/week	17.00 (9.37–30.37)	18.37 (9.62–34.25)	19.12 (10.50–35.00)	0.016 ^†^
AST, IU/L	21 (18–25)	21 (18–25)	21 (17–25)	0.488 ^†^
ALT, IU/L	16 (12–21)	16 (13–21)	17 (13–21))	0.078 ^†^
TG, mg/dL	106 (77–151)	111 (82–160)	120 (86.5–176.5)	<0.001 ^†^
T-Chol, mg/dL	189 (167–214)	192 (170–215)	191 (171–214)	0.097 ^†^
Albumin, g/dL	4.4 (4.2–4.6)	4.4 (4.3–4.6))	4.4 (4.2–4.6)	0.221 ^†^
Urine potassium, mmol/L	55 (35–82)	43 (30–64)	39 (27–55)	<0.001 ^†^
HOMA-IR	1.38 (1.02–1.95)	1.45 (1.03–2.06)	1.48 (1.05–2.08)	0.005 ^†^
eGFR, CKD-EPI, mL/min/1.73 m^2^	102.69 (93.11–110.03)	103.37 (94.73–110.51)	103.46 (95.80–109.84)	0.053 ^†^
Hypertension, *n* (%)	202 (23.46)	237 (27.53)	296 (34.42)	<0.001
Diabetes mellitus, *n* (%)	30 (3.48)	28 (3.25)	42 (4.88)	0.165
Hyperlipidaemia, *n* (%)	262 (29.31)	257 (28.78)	294 (32.92)	0.117
Postmenopausal, *n* (%)	290 (33.68)	312 (36.24)	350 (40.70)	0.005
Total energy, kcal	1796.7 (1484.2–2186.2)	1883.9 (1515.9–2325.8)	1930.1 (1592.8–2367.4)	<0.001 ^†^
Protein, g	58.92 (45.77–75.72)	62.08 (46.54–78.76)	63.77 (50.29–81.96)	<0.001 ^†^
Fat, g	27.04 (18.12–38.19)	27.57 (17.89–38.91)	29.05 (19.12–41.19)	0.002 ^†^
Carbohydrate, g	325.75 (273.96–388.04)	334.77 (280.48–405.66)	343.05 (290.38–422.54)	<0.001 ^†^

Continuous variables are presented as medians (interquartile ranges). Categorical variables are presented as numbers (%). Analysis of variance tests were used for continuous variables and Chi-squared test for categorical variables that were normally distributed. ^†^ Kruskal–Wallis test was performed for continuous variables. T1, lowest tertile, T2, middle tertile; T3, highest tertile; BMI, body mass index; eGFR, estimated glomerular filtration rate; CKD-EPI, chronic kidney disease epidemiology collaboration; HOMA-IR, homeostasis model assessment of insulin resistance; MET, metabolic equivalent of task; AST, aspartate aminotransferase; ALT, alanine aminotransferase; TG, triglyceride; T-Chol, total cholesterol.

**Table 3:** Hazard ratios (95% CI) for the incidence of NAFLD according to 24-h urinary sodium excretion.

	Hazard Ratio (95% CI)			*p*-Value
	T1	T2	T3	
All (*n* = 2582)	861	861	860	
Incident case, *n* (%)	139 (16.14)	182 (21.13)	203 (23.60)	<0.001
Crude analysis	Ref	1.32 (1.06, 1.65)	1.55 (1.25, 1.92)	<0.001 *
Multivariate analysis	Ref	1.31 (1.05–1.64)	1.54 (1.24–1.92)	<0.001 *
Men (*n* = 1011)	337	337	337	
Incident case, *n* (%)	49 (14.54)	58 (17.21)	66 (19.58)	0.174
Crude analysis	Ref	1.18 (0.80, 1.72)	1.41 (0.98, 2.04)	0.064 *
Multivariate analysis	Ref	1.19 (0.81, 1.75)	1.50 (1.02, 2.19)	<0.001 *
Females (*n* = 1571)	524	524	523	
Incident case, *n* (%)	90 (17.17)	124 (23.66)	137 (26.19)	0.001
Crude analysis	Ref	1.40 (1.07, 1.84)	1.62 (1.24, 2.12)	<0.001 *
Multivariate analysis	Ref	1.35 (1.03, 1.78)	1.51 (1.15, 1.98)	0.003 *

Multivariate analysis in all subjects: age, smoking status, triglyceride, total cholesterol, estimated glomerular filtration rate (eGFR), homeostatic model assessment of insulin resistance (HOMA-IR), hypertension, postmenopausal status. Multivariate analysis in males: age, physical activity, triglyceride, albumin, eGFR, and hyperlipidemia. Multivariate analysis in females: triglyceride, total cholesterol, albumin, HOMA-IR, and hypertension. * *p*-values were analyzed using the trend of the odds test. NAFLD, nonalcoholic fatty liver disease; CI, confidence interval; T1, lowest tertile; T2, middle tertile; T3, highest tertile.

**Table 4:** Hazard ratios (95% CI) for the incidence of hepatic fibrosis according to the 24-h urinary sodium excretion.

	Hazard Ratio (95% CI)			*p*-Value
	T1	T2	T3	
All (*n* = 2582)	861	861	860	
Incident case, *n* (%)	21 (2.43)	48 (5.57)	49 (5.69)	0.001
Crude analysis	Ref	2.25 (1.35, 3.77)	2.37 (1.42, 3.95)	0.001 *
Multivariate analysis	Ref	2.26 (1.35, 3.78)	2.28 (1.36, 3.83)	0.003 *
Men (*n* = 1011)	337	337	337	
Incident case, *n* (%)	7 (2.07)	17 (5.04)	18 (5.34)	0.178
Crude analysis	Ref	1.87 (0.83, 4.19)	2.04 (0.91, 4.55)	0.083 *
Multivariate analysis	Ref	1.93 (0.85, 4.36)	2.14 (0.95, 4.86)	0.073 *
Females (*n* = 1571)	524	524	523	
Incident case, *n* (%)	12 (1.90)	31 (4.77)	31 (5.73)	0.007
Crude analysis	Ref	2.54 (1.30, 4.95)	2.61 (1.34, 5.09)	0.005 *
Multivariate analysis	Ref	2.49 (1.27, 4.86)	2.42 (1.23, 4.73)	0.015 *

Multivariate analysis in all subjects: triglyceride, estimated glomerular filtration rate (eGFR), hypertension, postmenopausal status. Multivariate analysis in males: triglyceride, homeostatic model assessment of insulin resistance (HOMA-IR), eGFR, and hypertension. Multivariate analysis in females: triglyceride, eGFR, hypertension, and postmenopausal status. * *p*-values were analyzed using the trend of odds test. CI, confidence interval; T1, lowest tertile; T2, middle tertile; T3, highest tertile.

## Data Availability

This study used data from the Ansan–Ansung cohort in the Korean Genome Epidemiology Study (KoGES) which was conducted by National Institute of Health, Korea Disease Control and Prevention Agency, Ministry for Health and Welfare, Korea. The study data can be obtained upon request from the corresponding author and are not publicly accessible to safeguard personal information.

## Supplementary Materials

The following supporting information can be downloaded at: https://www.mdpi.com/article/10.3390/nu16040548/s1. Table S1. Baseline characteristics of male study participants (*n* = 1011) according to 24-h urinary sodium excretion. Table S2. Baseline characteristics of female study participants (*n* = 1571) according to 24-h urinary sodium excretion. Table S3. Risk factors for the incidence of NAFLD in all subjects (*n* = 2582) according to 24-h urinary sodium excretion. Table S4. Risk factors for the incidence of NAFLD in males (*n* = 1011) according to 24-h urinary sodium excretion. Table S5. Risk factors for the incidence of NAFLD in females (*n* = 1571) according to 24-h urinary sodium excretion. Table S6. Risk factors for the incidence of hepatic fibrosis in all subjects (*n* = 2582) according to 24-h urinary sodium excretion. Table S7. Risk factors for the incidence of hepatic fibrosis in males (*n* = 1011) according to 24-h urinary sodium excretion. Table S8. Risk factors for the incidence of hepatic fibrosis in females (*n* = 1571) according to 24-h urinary sodium excretion.

## References

[1] Pafili K., Maltezos E., Papanas N. (2018). Ipragliflozin and sodium glucose transporter 2 inhibitors to reduce liver fat: Will the prize we sought be won?. Expert. Opin. Pharmacother..

[2] Cusi K. (2016). Treatment of patients with type 2 diabetes and non-alcoholic fatty liver disease: Current approaches and future directions. Diabetologia.

[3] Younossi Z., Anstee Q.M., Marietti M., Hardy T., Henry L., Eslam M., George J., Bugianesi E. (2018). Global burden of NAFLD and NASH: Trends, predictions, risk factors and prevention. Nat. Rev. Gastroenterol. Hepatol..

[4] Pafili K., Roden M. (2021). Nonalcoholic fatty liver disease (NAFLD) from pathogenesis to treatment concepts in humans. Mol. Metab..

[5] Rinella M.E. (2015). Nonalcoholic fatty liver disease: A systematic review. JAMA.

[6] Weiss J., Rau M., Geier A. (2014). Non-alcoholic fatty liver disease: Epidemiology, clinical course, investigation, and treatment. Dtsch. Arztebl. Int..

[7] Dongiovanni P., M Anstee Q., Valenti L. (2013). Genetic predisposition in NAFLD and NASH: Impact on severity of liver disease and response to treatment. Curr. Pharm. Des..

[8] Zelber-Sagi S., Ratziu V., Oren R. (2011). Nutrition and physical activity in NAFLD: An overview of the epidemiological evidence. World J. Gastroenterol..

[9] Mouzaki M., Allard J.P. (2012). The role of nutrients in the development, progression, and treatment of nonalcoholic fatty liver disease. J. Clin. Gastroenterol..

[10] Schwarz J.M., Noworolski S.M., Wen M.J., Dyachenko A., Prior J.L., Weinberg M.E., Herraiz L.A., Tai V.W., Bergeron N., Bersot T.P. (2015). Effect of a High-Fructose Weight-Maintaining Diet on Lipogenesis and Liver Fat. J. Clin. Endocrinol. Metab..

[11] Wijarnpreecha K., Thongprayoon C., Edmonds P.J., Cheungpasitporn W. (2016). Associations of sugar- and artificially sweetened soda with nonalcoholic fatty liver disease: A systematic review and meta-analysis. QJM Int. J. Med..

[12] Bansal V., Mishra S.K. (2020). Reduced-sodium cheeses: Implications of reducing sodium chloride on cheese quality and safety. Compr. Rev. Food Sci. Food Saf..

[13] Campbell N.R.C., Whelton P.K., Orias M., Wainford R.D., Cappuccio F.P., Ide N., Neal B., Cohn J., Cobb L.K., Webster J. (2023). 2022 World Hypertension League, Resolve to Save Lives and International Society of Hypertension dietary sodium (salt) global call to action. J. Hum. Hypertens..

[14] Jeong Y., Kim E.S., Lee J., Kim Y. (2021). Trends in sodium intake and major contributing food groups and dishes in Korea: The Korea National Health and Nutrition Examination Survey 2013-2017. Nutr. Res. Pract..

[15] WHO (2012). Guideline: Sodium Intake for Adults and Children.

[16] Moosavian S.P., Haghighatdoost F., Surkan P.J., Azadbakht L. (2017). Salt and obesity: A systematic review and meta-analysis of observational studies. Int. J. Food Sci. Nutr..

[17] Baudrand R., Campino C., Carvajal C.A., Olivieri O., Guidi G., Faccini G., Vohringer P.A., Cerda J., Owen G., Kalergis A.M. (2014). High sodium intake is associated with increased glucocorticoid production, insulin resistance and metabolic syndrome. Clin. Endocrinol..

[18] Subasinghe A.K., Arabshahi S., Busingye D., Evans R.G., Walker K.Z., Riddell M.A., Thrift A.G. (2016). Association between salt and hypertension in rural and urban populations of low to middle income countries: A systematic review and meta-analysis of population based studies. Asia Pac. J. Clin. Nutr..

[19] Hu G., Jousilahti P., Peltonen M., Lindstrom J., Tuomilehto J. (2005). Urinary sodium and potassium excretion and the risk of type 2 diabetes: A prospective study in Finland. Diabetologia.

[20] Ha S.K. (2014). Dietary salt intake and hypertension. Electrolyte Blood Press..

[21] D’Elia L., Rossi G., Ippolito R., Cappuccio F.P., Strazzullo P. (2012). Habitual salt intake and risk of gastric cancer: A meta-analysis of prospective studies. Clin. Nutr..

[22] Huh J.H., Lee K.J., Lim J.S., Lee M.Y., Park H.J., Kim M.Y., Kim J.W., Chung C.H., Shin J.Y., Kim H.S. (2015). High Dietary Sodium Intake Assessed by Estimated 24-h urinary sodium Excretion Is Associated with NAFLD and Hepatic Fibrosis. PLoS ONE.

[23] Choi Y., Lee J.E., Chang Y., Kim M.K., Sung E., Shin H., Ryu S. (2016). Dietary sodium and potassium intake in relation to non-alcoholic fatty liver disease. Br. J. Nutr..

[24] Han E., Kim M.K., Im S.S., Kim H.S., Kwon T.K., Jang B.K. (2023). High Sodium Intake, as Assessed by Urinary sodium Excretion, Is Associated with Nonalcoholic Fatty Liver Disease or Sarcopenia. Gut Liver.

[25] Zhou L., Yang Y., Feng Y., Zhao X., Fan Y., Rong J., Zhao L., Yu Y. (2021). Association between dietary sodium intake and non-alcoholic fatty liver disease in the US population. Public Health Nutr..

[26] Kim Y., Han B.G., Ko G.E.S.g. (2017). Cohort Profile: The Korean Genome and Epidemiology Study (KoGES) Consortium. Int. J. Epidemiol..

[27] Tanaka T., Okamura T., Miura K., Kadowaki T., Ueshima H., Nakagawa H., Hashimoto T. (2002). A simple method to estimate populational 24-h urinary sodium and potassium excretion using a casual urine specimen. J. Hum. Hypertens..

[28] Lee J.H., Kim D., Kim H.J., Lee C.H., Yang J.I., Kim W., Kim Y.J., Yoon J.H., Cho S.H., Sung M.W. (2010). Hepatic steatosis index: A simple screening tool reflecting nonalcoholic fatty liver disease. Dig. Liver Dis..

[29] Sterling R.K., Lissen E., Clumeck N., Sola R., Correa M.C., Montaner J., Sulkowski S.M., Torriani F.J., Dieterich D.T., Thomas D.L. (2006). Development of a simple noninvasive index to predict significant fibrosis in patients with HIV/HCV coinfection. Hepatology.

[30] Levey A.S., Coresh J., Greene T., Stevens L.A., Zhang Y.L., Hendriksen S., Kusek J.W., Van Lente F., Chronic Kidney Disease Epidemiology C. (2006). Using standardized serum creatinine values in the modification of diet in renal disease study equation for estimating glomerular filtration rate. Ann. Intern. Med..

[31] Lefebvre P., Staels B. (2021). Hepatic sexual dimorphism—Implications for non-alcoholic fatty liver disease. Nat. Rev. Endocrinol..

[32] Ballestri S., Nascimbeni F., Baldelli E., Marrazzo A., Romagnoli D., Lonardo A. (2017). NAFLD as a Sexual Dimorphic Disease: Role of Gender and Reproductive Status in the Development and Progression of Nonalcoholic Fatty Liver Disease and Inherent Cardiovascular Risk. Adv. Ther..

[33] Lonardo A., Suzuki A. (2020). Sexual Dimorphism of NAFLD in Adults. Focus on Clinical Aspects and Implications for Practice and Translational Research. J. Clin. Med..

[34] Lee H.S., Duffey K.J., Popkin B.M. (2013). Sodium and potassium intake patterns and trends in South Korea. J. Hum. Hypertens..

[35] Shen X., Jin C., Wu Y., Zhang Y., Wang X., Huang W., Li J., Wu S., Gao X. (2019). Prospective study of perceived dietary salt intake and the risk of non-alcoholic fatty liver disease. J. Hum. Nutr. Diet..

[36] Van den Berg E.H., Gruppen E.G., Blokzijl H., Bakker S.J.L., Dullaart R.P.F. (2019). Higher Sodium Intake Assessed by 24 Hour Urinary sodium Excretion Is Associated with Non-Alcoholic Fatty Liver Disease: The PREVEND Cohort Study. J. Clin. Med..

[37] De Melo Portela C.L., de Carvalho Sampaio H.A., Pereira de Melo M.L., Ferreira Carioca A.A., Maia Pinto F.J., Machado Arruda S.P. (2015). Nutritional Status, Diet and Non-Alcoholic Fatty Liver Disease in Elders. Nutr. Hosp..

[38] Park S.H., Jeon W.K., Kim S.H., Kim H.J., Park D.I., Cho Y.K., Sung I.K., Sohn C.I., Keum D.K., Kim B.I. (2006). Prevalence and risk factors of non-alcoholic fatty liver disease among Korean adults. J. Gastroenterol. Hepatol..

[39] Eguchi Y., Hyogo H., Ono M., Mizuta T., Ono N., Fujimoto K., Chayama K., Saibara T., Jsg N. (2012). Prevalence and associated metabolic factors of nonalcoholic fatty liver disease in the general population from 2009 to 2010 in Japan: A multicenter large retrospective study. J. Gastroenterol..

[40] McKenzie J., Fisher B.M., Jaap A.J., Stanley A., Paterson K., Sattar N. (2006). Effects of HRT on liver enzyme levels in women with type 2 diabetes: A randomized placebo-controlled trial. Clin. Endocrinol..

[41] Grillo A., Salvi L., Coruzzi P., Salvi P., Parati G. (2019). Sodium Intake and Hypertension. Nutrients.

[42] Lastra G., Dhuper S., Johnson M.S., Sowers J.R. (2010). Salt, aldosterone, and insulin resistance: Impact on the cardiovascular system. Nat. Rev. Cardiol..

[43] Rinaldi L., Pafundi P.C., Galiero R., Caturano A., Morone M.V., Silvestri C., Giordano M., Salvatore T., Sasso F.C. (2021). Mechanisms of Non-Alcoholic Fatty Liver Disease in the Metabolic Syndrome. A Narrative Review. Antioxidants.

[44] Lee J.H., Lee H.S., Ahn S.B., Kwon Y.J. (2021). Dairy protein intake is inversely related to development of non-alcoholic fatty liver disease. Clin. Nutr..

[45] Nyblom H., Berggren U., Balldin J., Olsson R. (2004). High AST/ALT ratio may indicate advanced alcoholic liver disease rather than heavy drinking. Alcohol Alcohol..

