# Supplementary material for: Sex-Specific Association between Sodium Intake Estimated by 24-Hour Urinary Sodium Excretion and Nonalcoholic Fatty Liver Disease: The Community-Based Prospective Cohort Study
Source: Nutrients. 2024 Feb 16;16(4):548. doi: 10.3390/nu16040548 (PMC10892959; doi:10.3390/nu16040548)
Supplement: Supplementary file 1 [file nutrients-16-00548-s001.zip › nutrients-2865483-supplementary.pdf]

Supplementary Information.

Sex–specific association between sodium intake assessed by 24-h urinary sodium excretion and nonalcoholic fatty liver disease: the Korean Genome and Epidemiology Study

Jihye Lee[1†], Ju–Yeon Lee[2†], Yun–Jung Yang[3*]

[1]Occupational Safety and Health Research Institute, Korea Occupational Safety and Health Agency, Ulsan 44429, Republic of Korea
[2]College of Medicine, Catholic Kwandong University, Gangneung–si 25601, Republic of Korea
[3]Department of Convergence Science, College of Medicine, Catholic Kwandong University International St. Mary's Hospital, Incheon 22711, Republic of Korea

* Correspondence: yangyj@ish.ac.kr

†These authors contributed equally to this study.

Table S1. Baseline characteristics of male study participants (n = 1011) according to 24-hour urinary sodium excretion

Characteristics	Tertiles of 24-hour urinary sodium excretion (mmol/day)			p-Value
	T1 (n = 337)	T2 (n = 337)	T3 (n = 337)	
24-hour urinary sodium, mmol/day	129.63 (117.16–138.36)	160.99 (153.54–168.07)	192.54 (182.89–206.84)	< 0.001†
Age, yr	51 (44–59)	52 (45–61)	53 (46–62)	0.002†
BMI, kg/m^2	22.82 (21.12–24.56)	23.29 (21.77–24.97)	23.62 (22.01–25.44)	< 0.001†
Drinking status, n (%)				0.549
Never	87 (25.82)	88 (26.11)	85 (25.22)	
Former	42 (12.46)	54 (16.02)	41 (12.17)	
Current	208 (61.72)	195 (57.86)	211 (62.61)	
Smoking status, n (%)				< 0.001
Never	58 (17.21)	81 (24.04)	97 (28.78)	
Former	78 (23.15)	89 (26.41)	99 (29.38)	
Current	201 (59.64)	167 (49.55)	141 (41.84)	
Physical activity, MET–h/week	17.87 (8.25–36.87)	20.25 (9.75–36.87)	20.37 (9.75–36.37)	0.173†
AST, IU/L	23 (19–28)	22 (19–27)	22 (20–27)	0.486†
ALT, IU/L	20 (16–27)	20 (15–25)	20 (16–25)	0.283†
TG, mg/dL	122 (88–173)	130 (93–180)	133 (96–203)	0.014†
T–Chol, mg/dL	192 (168–213)	192 (168–213)	186 (167–208)	0.114†
Albumin, g/dL	4.5 (4.3–4.7)	4.5 (4.3–4.6)	4.5 (4.3–4.7)	0.069†
Urine potassium, mmol/L	59 (39–90)	44 (32–66)	41 (28–57)	< 0.001†
HOMA–IR	1.33 (0.96–1.74)	1.30 (0.92–1.90)	1.38 (0.96–2.00)	0.110†
eGFR, CKD–EPI, l/min/1.73m^2	99.61 (87.02–107.79)	100.99 (93.41–108.14)	102.89 (94.78–108.48)	< 0.001†
Hypertension, n (%)	88 (26.11)	100 (29.67)	131 (38.87)	0.001
Diabetes mellitus, n (%)	15 (4.45)	18 (5.34)	25 (7.42)	0.236
Hyperlipidemia, n (%)	181 (53.71)	188 (55.79)	175 (51.93)	0.603
Total energy, kcal	1893.2 (1581.7–2249.5)	1969.4 (1595.7–2374.7)	2047.5 (1696.7–2627.3)	< 0.001†
Protein, g	61.32 (47.74–76.73)	64.27 (50.90–82.11)	67.98 (54.07–88.44)	< 0.001†
Fat, g	30.17 (20.48–41.43)	31.40 (21.08–41.81)	33.47 (22.29–47.43)	0.001†

Carbohydrate, g	338.34 (285.97–393.97)	345.34 (286.01–415.32)	355.03 (303.81–448.06)	< 0.001†

Continuous variables are presented as medians (interquartile ranges). Categorical variables are presented as numbers (%). Analysis of variance tests were used for continuous variables and Chi–squared test for categorical variables that were normally distributed. † Kruskal–Wallis test was performed for continuous variables.

T1, lowest tertile; T2, middle tertile; T3, highest tertile; BMI, body mass index; eGFR, estimated glomerular filtration rate; CKD–EPI, chronic kidney disease epidemiology collaboration; HOMA–IR, homeostasis model assessment of insulin resistance; MET, metabolic equivalent of task; AST, aspartate aminotransferase; ALT, alanine aminotransferase; TG, triglyceride; T–Chol, total cholesterol

Table S2. Baseline characteristics of female study participants (n = 1571) according to 24-h urinary sodium excretion

Characteristics	Tertiles of 24-hour urinary sodium excretion (mmol/day)			p-Value
	T1 (n = 524)	T2 (n = 524)	T3 (n = 523)	
24-hour urinary sodium, mmol/day	129.03 (114.40–138.75)	160.20 (152.68–166.82)	193.14 (182.58–210.16)	< 0.001†
Age, yr	48 (43–59)	50 (44–61)	53 (45–61)	< 0.001†
BMI, kg/m²	23.07 (21.51–24.65)	23.69 (21.94–25.05)	24.02 (22.74–25.30)	< 0.001†
Drinking status, n (%)				0.438
Never	402 (76.72)	384 (73.28)	402 (76.86)	
Former	16 (3.05)	12 (2.29)	13 (2.49)	
Current	106 (20.23)	128 (24.43)	108 (20.65)	
Smoking status, n (%)				0.669
Never	497 (94.85)	504 (96.18)	505 (96.56)	
Former	4 (0.76)	4 (0.76)	3 (0.57)	
Current	23 (4.39)	16 (3.05)	15 (2.87)	
Physical activity, MET–h/week	16.43 (9.81–27.25)	17.00 (9.37–30.18)	18.75 (11.00–31.62)	0.029†
AST, IU/L	19 (17–23)	20 (17–24)	19 (17–23)	0.414†
ALT, IU/L	14 (12–18)	15 (12–19)	15 (12–18)	0.028†
TG, mg/dL	96.5 (72–135)	103.5 (76–148)	115 (82–162)	< 0.001†
T–Chol, mg/dL	187 (154–214.5)	192 (171–215.5)	193 (173–219)	0.004†
Albumin, g/dL	4.4 (4.2–4.5)	4.4 (4.2–4.5)	4.4 (4.2–4.5)	0.413†
Urine potassium, mmol/L	53 (32–77)	43 (30–64)	37 (26–55)	< 0.001†
HOMA–IR	1.43 (1.06–2.02)	1.55 (1.09–2.13)	1.53 (1.12–2.12)	0.016†
eGFR, CKD–EPI, ml/min/1.73m²	104.65 (94.80–112.03)	105.28 (95.32–111.37)	103.99 (97.10–110.69)	0.366
Hypertension, n (%)	114 (21.76)	137 (26.15)	165 (31.55)	0.002
Diabetes mellitus, n (%)	15 (2.86)	10 (1.91)	17 (3.25)	0.383
Hyperlipidemia, n (%)	256 (48.85)	254 (48.47)	259 (49.52)	0.943
Postmenopausal, n (%)	290 (55.34)	312 (59.54)	350 (66.92)	0.001
Total energy, kcal	1730.8 (1423.9–2139.0)	1841.8 (1483.5–2273.0)	1870.2 (1540.4–2244.6)	0.062
Protein, g	57.53 (43.50–74.45)	60.24 (44.81–77.79)	60.84 (47.91–77.68)	0.005†

Fat, g	25.17 (16.81–36.07)	25.55 (16.94–36.93)	25.53 (17.81–36.48)	0.104†
Carbohydrate, g	313.41 (260.77–384.44)	328.79 (275.60–399.83)	337.36 (284.89–405.91)	< 0.001†

Continuous variables are presented as medians (interquartile ranges). Categorical variables are presented as numbers (%). Analysis of variance tests were used for continuous variables and Chi–squared test for categorical variables that were normally distributed. † Kruskal–Wallis test was performed for continuous variables.

T1, lowest tertile; T2, middle tertile; T3, highest tertile; BMI, body mass index; eGFR, estimated glomerular filtration rate; CKD–EPI, chronic kidney disease epidemiology collaboration; HOMA–IR, homeostasis model assessment of insulin resistance; MET, metabolic equivalent of task; AST, aspartate aminotransferase; ALT, alanine aminotransferase; TG, triglyceride; T–Chol, total cholesterol

Table S3. Risk factors for the incidence of NAFLD in all subjects (n = 2582) according to 24-hour urinary sodium excretion

	Univariate		Multivariate	
	**HR (95% CI)**	***p*-Value**	**HR (95% CI)**	***p*-Value**
Age, yr	0.98 (0.97–0.99)	0.002	0.96 (0.94–0.97)	< 0.001
BMI, kg/m^2	1.64 (1.56–1.72)	< 0.001	–	–
Drinking status, *n* (%)				
Never	Ref		–	–
Former	0.81 (0.56–1.19)	0.296	–	–
Current	0.89 (0.74–1.06)	0.213	–	–
Smoking status, *n* (%)				
Never	Ref		Ref	
Former	0.85 (0.64–1.13)	0.289	0.92 (0.63–1.34)	0.677
Current	0.71 (0.56–0.90)	0.005	0.89 (0.64–1.22)	0.482
Physical activity, MET–h/week	0.99 (0.99–1.00)	0.624	–	–
TG, mg/dL	1.00 (1.00–1.00)	< 0.001	1.00 (1.00–1.00)	< 0.001
T–Chol, mg/dL	1.00 (1.00–1.00)	0.004	1.00 (0.99–1.00)	0.612
Albumin, g/dL	1.52 (1.07–2.18)	0.019	1.21 (0.81–1.81)	0.349
Urine potassium, mmol/L	1.00 (0.99–1.00)	0.746	–	–
HOMA–IR	1.08 (1.03–1.13)	< 0.001	1.07 (1.02–1.12)	0.006
eGFR, CKD–EPI, ml/min/1.73m^2	0.99 (0.98–0.99)	0.016	0.98 (0.97–0.99)	< 0.001
Hypertension, *n* (%)	1.25 (1.04–1.50)	0.017	1.30 (1.07–1.59)	0.007
Diabetes mellitus, *n* (%)	2.23 (1.57–3.16)	< 0.001	–	–
Hyperlipidemia, *n* (%)	1.24 (1.04–1.48)	0.012	1.05 (0.84–1.31)	0.634
Postmenopausal, *n* (%)	0.96 (0.77–1.19)	0.737	1.10 (0.83–1.45)	0.489
Total energy, kcal	0.99 (0.99–1.00)	0.377	–	–
Protein, g	0.99 (0.99–1.00)	0.586	–	–
Fat, g	0.99 (0.99–1.00)	0.491	–	–
Carbohydrate, g	0.99 (0.99–1.00)	0.412	–	–

Variables with p-values < 0.10 in the univariate analysis were included in the multivariate analysis. BMI and DM were not considered in multivariate analysis because they were used for NAFLD classification.

NAFLD, nonalcoholic fatty liver disease; HR, hazard ratio; CI, confidence interval; BMI, body mass index; eGFR, estimated glomerular filtration rate; CKD–EPI, chronic kidney disease epidemiology collaboration; HOMA–IR, homeostasis model assessment of insulin resistance; MET, metabolic equivalent of task; AST, aspartate aminotransferase; ALT, alanine aminotransferase; TG, triglyceride; T–Chol, total cholesterol

Table S4. Risk factors for the incidence of NAFLD in males (n = 1011) according to 24-hour urinary sodium excretion

	**Univariate**		**Multivariate**	
	**HR (95% CI)**	***p*-Value**	**HR (95% CI)**	***p*-Value**
Age, yr	0.96 (0.94–0.98)	< 0.001	0.95 (0.93–0.97)	< 0.001
BMI, kg/m^2	1.62 (1.50–1.74)	< 0.001	–	–
Drinking status, *n* (%)				
Never	Ref		–	–
Former	0.94 (0.57–1.56)	0.831	–	–
Current	0.95 (0.67–1.34)	0.779	–	–
Smoking status, *n* (%)				
Never	Ref		–	–
Former	1.24 (0.82–1.89)	0.299	–	–
Current	1.04 (0.71–1.54)	0.810	–	–
Physical activity, MET–h/week	0.99 (0.98–1.00)	0.156	-	-
TG, mg/dL	1.00 (1.00–1.00)	< 0.001	1.00 (1.00–1.00)	0.002
T–Chol, mg/dL	1.00 (0.99–1.00)	0.055	1.00 (0.99–1.00)	0.574
Albumin, g/dL	2.38 (1.31–4.30)	0.004	1.13 (0.57–2.22)	0.713
Urine potassium, mmol/L	0.99 (0.99–1.00)	0.898	–	–
HOMA–IR	1.12 (1.03–1.21)	0.004	1.12 (1.02–1.23)	0.013
eGFR, CKD–EPI, ml/min/1.73m^2	0.98 (0.97–1.00)	0.063	0.97 (0.96–0.99)	< 0.001
Hypertension, *n* (%)	1.21 (0.88–1.66)	0.227	–	–
Diabetes mellitus, *n* (%)	2.93 (1.85–4.62)	< 0.001	–	–
Hyperlipidemia, *n* (%)	1.36 (1.00–1.84)	0.047	1.02 (0.69–1.49)	0.912
Total energy, kcal	1.00 (0.99–1.00)	0.901	–	–
Protein, g	1.00 (0.99–1.00)	0.744	–	–
Fat, g	0.99 (0.99–1.00)	0.886	–	–
Carbohydrate, g	1.00 (0.99–1.00)	0.768	–	–

Variables with p-values < 0.10 in the univariate analysis were included in the multivariate analysis. BMI and DM were not considered in multivariate analysis because they were used for NAFLD classification.

NAFLD, nonalcoholic fatty liver disease; HR, hazard ratio; CI, confidence interval; BMI, body mass index; eGFR, estimated glomerular filtration rate; CKD–EPI, chronic kidney disease epidemiology collaboration; HOMA–IR, homeostasis model assessment of insulin resistance; MET, metabolic equivalent of task; AST, aspartate aminotransferase; ALT, alanine aminotransferase; TG, triglyceride; T–Chol, total cholesterol

Table S5. Risk factors for the incidence of NAFLD in females (*n* = 1571) according to 24-hour urinary sodium excretion

	**Univariate**		**Multivariate**	
	**HR (95% CI)**	***p*-Value**	**HR (95% CI)**	***p*-Value**
Age, yr	0.99 (0.98–1.00)	0.417	–	–
BMI, kg/m^2	1.66 (1.57–1.77)	< 0.001	–	–
Drinking status, *n* (%)				
Never	Ref		–	–
Former	0.97 (0.45–2.05)	0.938	–	–
Current	1.05 (0.82–1.35)	0.681	–	–
Smoking status, *n* (%)				
Never	Ref		Ref	–
Former	0.38 (0.05–2.72)	0.337	0.42 (0.05–2.99)	0.387
Current	0.43 (0.18–1.05)	0.067	0.42 (0.17–1.03)	0.060
Physical activity, MET–h/week	1.00 (0.99–1.00)	0.408	–	–
TG, mg/dL	1.00 (1.00–1.00)	< 0.001	1.00 (1.00–1.00)	0.005
T–Chol, mg/dL	1.00 (0.99–1.00)	0.041	1.00 (0.99–1.00)	0.983
Albumin, g/dL	1.60 (0.99–2.58)	0.055	1.37 (0.83–2.26)	0.205
Urine potassium, mmol/L	1.00 (0.99–1.00)	0.422	–	–
HOMA–IR	1.06 (1.01–1.12)	0.019	1.04 (0.98–1.10)	0.120
eGFR, CKD–EPI, ml/min/1.73m^2	0.99 (0.98–0.99)	0.033	0.99 (0.98–1.00)	0.136
Hypertension, *n* (%)	1.30 (1.03–1.63)	0.023	1.12 (0.88–1.42)	0.334
Diabetes mellitus, *n* (%)	1.85 (1.06–3.22)	0.030	–	–
Hyperlipidemia, *n* (%)	1.21 (0.98–1.50)	0.063	1.03 (0.78–1.36)	0.819
Postmenopausal, *n* (%)	0.96 (0.78–1.19)	0.749	–	–
Total energy, kcal	0.99 (0.99–1.00)	0.426	–	–
Protein, g	0.99 (0.99–1.00)	0.559	–	–
Fat, g	0.99 (0.99–1.00)	0.812	–	–
Carbohydrate, g	0.99 (0.99–1.00)	0.419	–	–

Variables with *p*-values < 0.10 in the univariate analysis were included in the multivariate analysis. BMI and DM were not considered in multivariate analysis because they were used for NAFLD classification.

NAFLD, nonalcoholic fatty liver disease; HR, hazard ratio; CI, confidence interval; BMI, body mass index; eGFR, estimated glomerular filtration rate; CKD–EPI, chronic kidney disease epidemiology collaboration; HOMA–IR, homeostasis model assessment of insulin resistance; MET, metabolic equivalent of task; AST, aspartate aminotransferase; ALT, alanine aminotransferase; TG, triglyceride; T–Chol, total cholesterol

Table S6. Risk factors for the incidence of hepatic fibrosis in all subjects (*n* = 2582) according to 24-hour urinary sodium excretion

	**Univariate**		**Multivariate**	
	**HR (95% CI)**	***p*-Value**	**HR (95% CI)**	***p*-Value**
Age, yr	1.06 (1.04–1.08)	< 0.001	–	–
Gender (women), *n* (%)	1.04 (0.72-1.51)	0.814		
BMI, kg/m^2	1.64 (1.49–1.80)	< 0.001	–	–
Drinking status, *n* (%)				
Never	Ref		–	–
Former	1.23 (0.61–2.47)	0.560	–	–
Current	1.09 (0.72–1.55)	0.756	–	–
Smoking status, *n* (%)				
Never	Ref		–	–
Former	1.24 (0.72–2.12)	0.427	–	–
Current	0.81 (0.50–1.32)	0.414	–	–
Physical activity, MET–h/week	0.99 (0.98–1.01)	0.538	–	–
TG, mg/dL	1.00 (1.00–1.00)	0.001	1.00 (1.00–1.00)	0.035
T–Chol, mg/dL	1.00 (0.99–1.01)	0.520	–	–
Albumin, g/dL	0.88 (0.41–1.85)	0.738	–	–
Urine potassium, mmol/L	1.00 (0.99–1.01)	0.178	–	–
HOMA–IR	1.10 (1.01–1.19)	0.016	–	–
eGFR, CKD–EPI, ml/min/1.73m^2	0.96 (0.95–0.97)	< 0.001	0.96 (0.95–0.98)	< 0.001
Hypertension, *n* (%)	1.97 (1.36–2.83)	<0.001	1.53 (1.05–2.23)	0.026
Diabetes mellitus, *n* (%)	2.71 (1.41–5.18)	0.003	–	–
Hyperlipidemia, *n* (%)	1.18 (0.82–1.70)	0.363	–	–
Postmenopausal, *n* (%)	2.32 (1.35–3.99)	0.002	1.36 (0.77–2.40)	0.288
Total energy, kcal	1.00 (0.99–1.00)	0.400	–	–
Protein, g	1.00 (0.99–1.00)	0.542	–	–
Fat, g	1.00 (0.99–1.00)	0.914	–	–
Carbohydrate, g	1.00 (0.99–1.00)	0.298	–	–

Variables with *p*-values < 0.10 in the univariate analysis were included in the multivariate analysis. Age, BMI, and DM were not considered in multivariate analysis because they were used for NAFLD and hepatic fibrosis classification.

HR, hazard ratio; CI, confidence interval; BMI, body mass index; eGFR, estimated glomerular filtration rate; CKD–EPI, chronic kidney disease epidemiology collaboration; HOMA–IR, homeostasis model assessment of insulin resistance; MET, metabolic equivalent of task; AST, aspartate aminotransferase; ALT, alanine aminotransferase; TG, triglyceride; T–Chol, total cholesterol

Table S7. Risk factors for the incidence of hepatic fibrosis in males (n = 1011) according to 24-hour urinary sodium excretion

	Univariate		Multivariate	
	**HR (95% CI)**	***p*-Value**	**HR (95% CI)**	***p*-Value**
Age, yr	1.07 (1.03–1.11)	< 0.001	–	–
BMI, kg/m^2	1.64 (1.46–1.91)	< 0.001	–	–
Drinking status, *n* (%)				
Never	Ref		–	–
Former	1.87 (0.74–4.73)	0.181	–	–
Current	1.19 (0.55–2.54)	0.651	–	–
Smoking status, *n* (%)				
Never	Ref		–	–
Former	1.41 (0.63–3.10)	0.394	–	–
Current	0.87 (0.40–1.89)	0.739	–	–
Physical activity, MET–h/week	0.98 (0.97–1.00)	0.225	–	–
TG, mg/dL	1.00 (0.99–1.00)	0.006	1.00 (1.00–1.00)	0.024
T–Chol, mg/dL	1.00 (0.99–1.01)	0.912	–	–
Albumin, g/dL	0.45 (0.14–1.42)	0.175	–	–
Urine potassium, mmol/L	1.00 (0.99–1.01)	0.272	–	–
HOMA–IR	1.17 (1.03–1.33)	0.014	1.14 (0.98-1.33)	0.076
eGFR, CKD–EPI, ml/min/1.73m^2	0.96 (0.94–0.98)	<0.001	0.95 (0.93–0.97)	< 0.001
Hypertension, *n* (%)	1.73 (0.95–3.15)	0.069	1.46 (0.80-2.68)	0.211
Diabetes mellitus, *n* (%)	4.94 (2.37–10.29)	< 0.001	–	–
Hyperlipidemia, *n* (%)	1.20 (0.65–2.18)	0.551	–	–
Total energy, kcal	0.99 (0.99–1.00)	0.494	–	–
Protein, g	0.99 (0.98–1.01)	0.640	–	–
Fat, g	0.99 (0.97–1.01)	0.536	–	–
Carbohydrate, g	0.99 (0.99–1.00)	0.602	–	–

Variables with *p*-values < 0.10 in the univariate analysis were included in the multivariate analysis. Age, BMI, and DM were not considered in multivariate analysis because they were used for NAFLD and hepatic fibrosis classification.

HR, hazard ratio; CI, confidence interval; BMI, body mass index; eGFR, estimated glomerular filtration rate; CKD–EPI, chronic kidney disease epidemiology collaboration; HOMA–IR, homeostasis model assessment of insulin resistance; MET, metabolic equivalent of task; AST, aspartate aminotransferase; ALT, alanine aminotransferase; TG, triglyceride; T–Chol, total cholesterol

Table S8. Risk factors for the incidence of hepatic fibrosis in females (n = 1571) according to 24-hour urinary sodium excretion

	**Univariate**		**Multivariate**	
	**HR (95% CI)**	***p*-Value**	**HR (95% CI)**	***p*-Value**
Age, yr	1.05 (1.03–1.08)	< 0.001	–	–
BMI, kg/m^2	1.61 (1.42–1.82)	< 0.001	–	–
Drinking status, *n* (%)				
Never	Ref		–	–
Former	–	–	–	–
Current	1.16 (0.68–1.95)	0.573	–	–
Smoking status, *n* (%)				
Never	Ref		–	–
Former	–	–	–	–
Current	0.94 (0.23-3.84)	0.934	–	–
Physical activity, MET–h/week	1.00 (0.98–1.01)	0.806	–	–
TG, mg/dL	1.00 (0.99–1.00)	0.089	1.00 (0.99–1.00)	0.857
T–Chol, mg/dL	1.00 (0.99–1.00)	0.474	–	–
Albumin, g/dL	1.57 (0.55–4.44)	0.395	–	–
Urine potassium, mmol/L	1.00 (0.99–1.01)	0.383	–	–
HOMA–IR	1.07 (0.96–1.19)	0.205	–	–
eGFR, CKD–EPI, ml/min/1.73m^2	0.97 (0.95–0.98)	<0.001	0.97 (0.96–0.99)	0.006
Hypertension, *n* (%)	2.15 (1.35–3.41)	0.001	1.66 (1.02–2.69)	0.041
Diabetes mellitus, *n* (%)	0.58 (0.08-4.19)	0.592	–	–
Hyperlipidemia, *n* (%)	1.18 (0.75–1.87)	0.463	–	–
Postmenopausal, *n* (%)	2.36 (1.37–4.05)	0.002	1.54 (0.85–2.78)	0.150
Total energy, kcal	1.00 (0.99–1.00)	0.125	–	–
Protein, g	1.00 (0.98–1.00)	0.303	–	–
Fat, g	1.00 (0.99–1.01)	0.551	–	–
Carbohydrate, g	1.00 (0.99–1.00)	0.094	–	–

Variables with p-values < 0.10 in the univariate analysis were included in the multivariate analysis. Age, BMI, and DM were not considered in multivariate analysis because they were used for NAFLD and hepatic fibrosis classification.

HR, hazard ratio; CI, confidence interval; BMI, body mass index; eGFR, estimated glomerular filtration rate; CKD–EPI, chronic kidney disease epidemiology collaboration; HOMA–IR, homeostasis model assessment of insulin resistance; MET, metabolic equivalent of task; AST, aspartate aminotransferase; ALT, alanine aminotransferase; TG, triglyceride; T–Chol, total cholesterol
